# Author Correction: A mechanistic framework for auxin dependent *Arabidopsis* root hair elongation to low external phosphate

**DOI:** 10.1038/s41467-018-04281-x

**Published:** 2018-05-02

**Authors:** Rahul Bhosale, Jitender Giri, Bipin K. Pandey, Ricardo F. H. Giehl, Anja Hartmann, Richard Traini, Jekaterina Truskina, Nicola Leftley, Meredith Hanlon, Kamal Swarup, Afaf Rashed, Ute Voß, Jose Alonso, Anna Stepanova, Jeonga Yun, Karin Ljung, Kathleen M. Brown, Jonathan P. Lynch, Liam Dolan, Teva Vernoux, Anthony Bishopp, Darren Wells, Nicolaus von Wirén, Malcolm J. Bennett, Ranjan Swarup

**Affiliations:** 1 Plant & Crop Sciences, School of Biosciences, University of Nottingham, Nottingham, LE12 5RD UK; 2 Centre for Plant Integrative Biology (CPIB), University of Nottingham, Nottingham, LE12 5RD UK; 3 National Institute of Plant Genome Research (NIPGR), New Delhi, 110067 India; 4 Leibniz Institute of Plant Genetics and Crop Plant Research (IPK), D-06466 OT Gatersleben, Stadt Seeland Germany; 5 Laboratoire Reproduction et Développement des Plantes, Univ Lyon, ENS de Lyon, UCB Lyon 1, CNRS, INRA, F-69342 Lyon, France; 6 Department of Plant Science, The Pennsylvania State University, 102 Tyson Building, University Park, PA 16802 USA; 7 Department of Plant and Microbial Biology, NC State University, Raleigh, NC 27695 USA; 8 Umeå Plant Science Centre, Department of Forest Genetics and Plant Physiology, Swedish University of Agricultural Sciences, SE-901 83 Umeå, Sweden; 9 Department of Plant Sciences, University of Oxford, Oxford, OX1 3RB UK

Correction to: *Nature Communications* 10.1038/s41467-018-03851-3, published online 12 April 2018

The original version of this Article omitted the following from the Acknowledgements:

‘We also thank DBT-CREST BT/HRD/03/01/2002.’

This has been corrected in both the PDF and HTML versions of the Article.

