# Supplementary material for: A mechanistic framework for auxin dependent Arabidopsis root hair elongation to low external phosphate
Source: Nat Commun. 2018 Apr 12;9:1409. doi: 10.1038/s41467-018-03851-3 (PMC5897496; doi:10.1038/s41467-018-03851-3)
Supplement: Supplementary file 1 — Supplementary Information [file 41467_2018_3851_MOESM1_ESM.pdf]

A mechanistic framework for auxin dependent *Arabidopsis* root hair elongation to low external phosphate

Bhosale et al

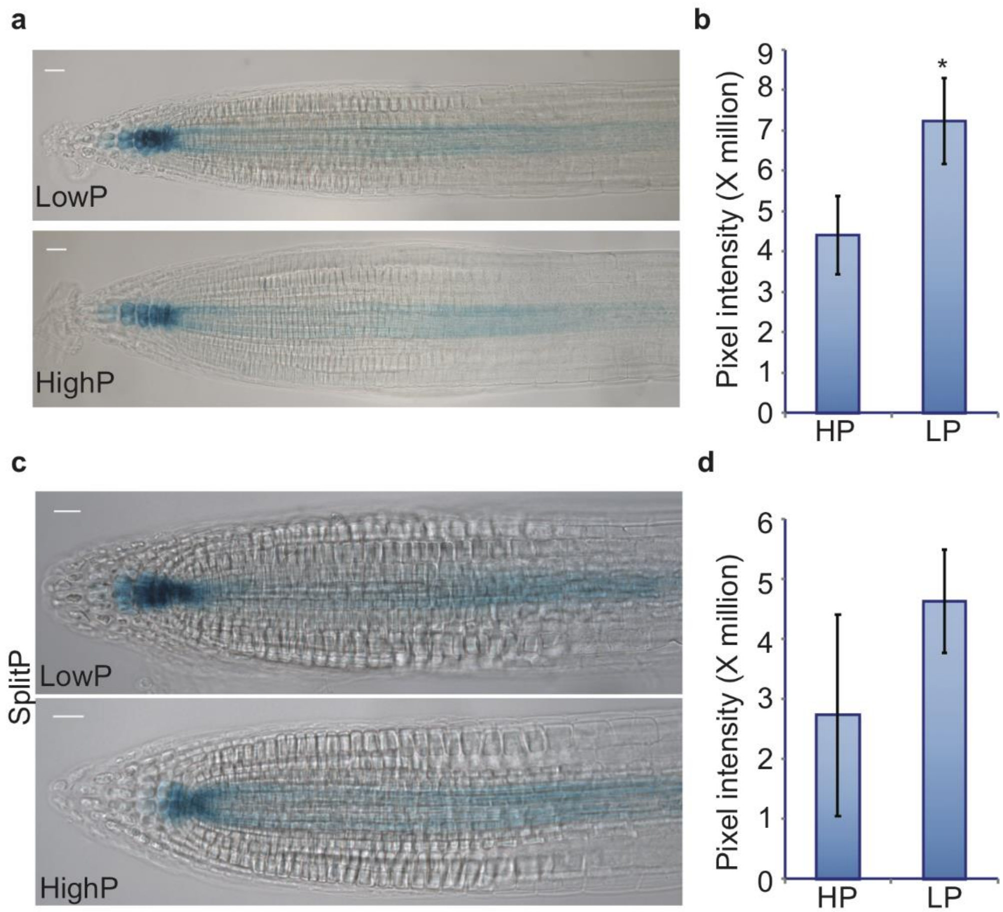

Supplementary Figure 1. TAA1-GUS assays reveal that *TAA1* expression is up regulated under low P conditions.

a & b. Five day old TAA1::GUS seedlings were transferred to low P (LP) or high P (HP) media and the GUS staining was done 1 day after the transfer (a) and GUS signal intensity quantified using image J (b). Scale bar 20 µm. *Indicate significant difference q-value < 0.005, judged by Student's *t*-test.

c & d. Split P experiment: Primary roots of the two day old TAA1::GUS seedlings were excised and the resulting anchor roots (3 days after the excision) were split onto either LP or HP media. GUS staining was done 1 day after the transfer (c) and GUS signal intensity quantified using image J (d). At least 5 independent roots were analysed to quantify the GUS expression in each condition. Scale bar 20 µm.

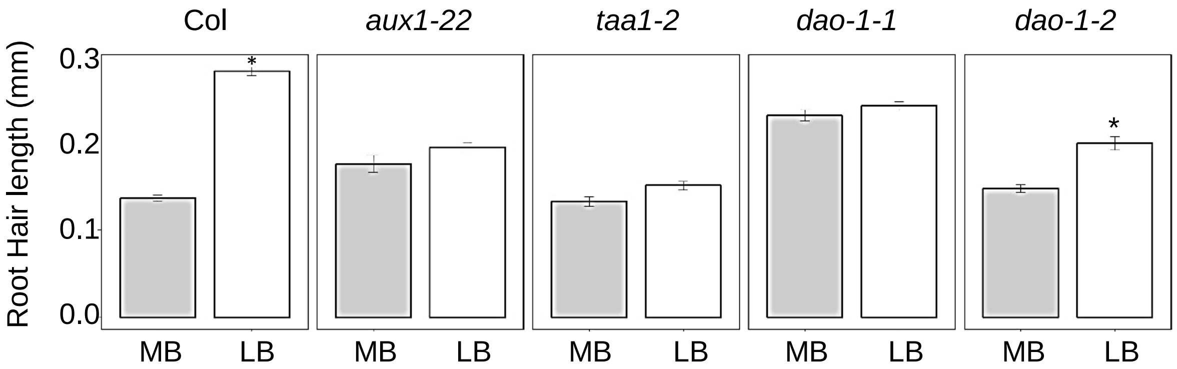

Supplementary Figure 2. Low P adaptive root hair elongation in buffered nutrient system

Root hair length of plants grown under sufficient (medium P buffered MB, 50 µM soluble P) and limiting (Low P buffered LB, 3µM soluble P) phosphorus conditions with a buffered phosphorus delivery regime. Plants were grown for three days (16h day, 100-120 µmol min^{-2}) on ¼ strength MS media without sucrose before being transferred to the respective treatment. After four days of growth, images were obtained on a dissecting scope and ten root hairs were imaged. Ten plants per genotype, per treatment were measured. Error bars represent the standard error of the mean. *indicates significant difference (p value <0.01 Welch Two Sample t-test)

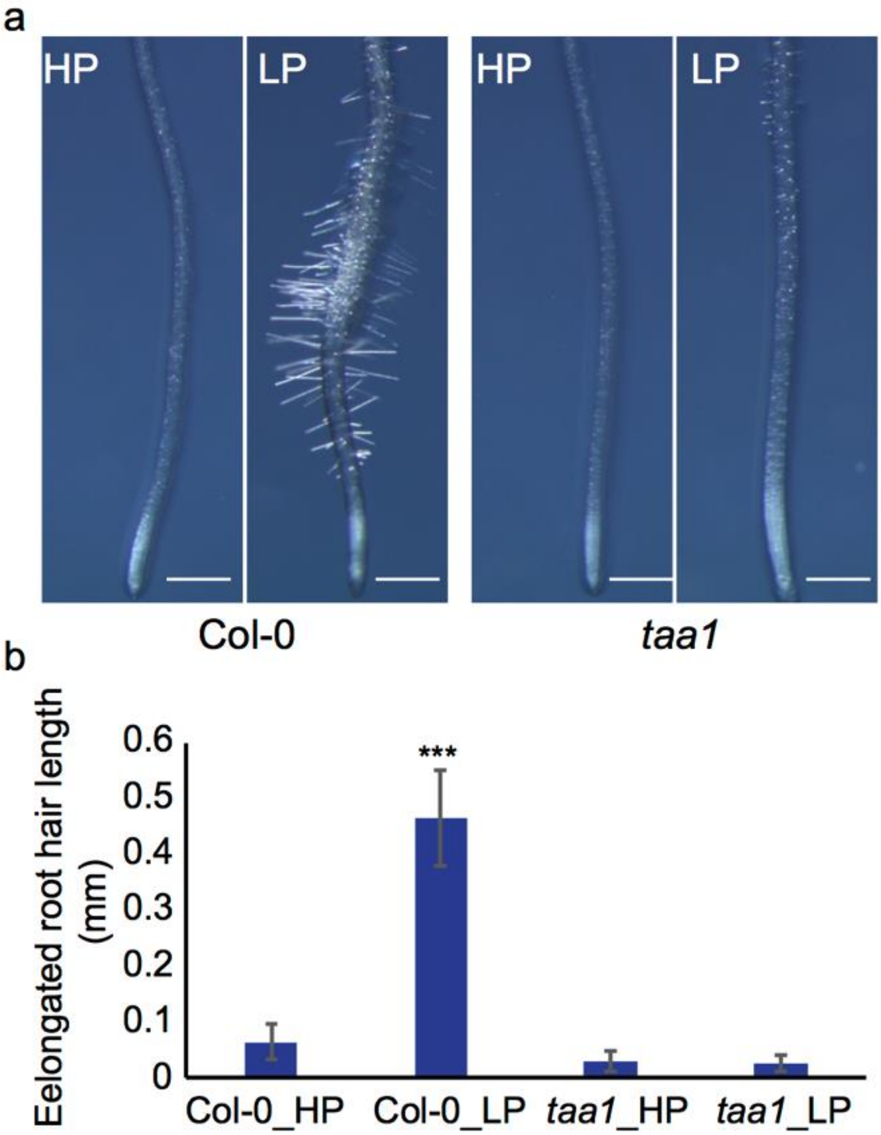

Supplementary Figure 3. TAA1 is required for low P mediated root hair elongation
Two day old Columbia (Col-0) and taa1 seedling primary roots were excised to induce anchor roots. After three days resulting anchor roots of the same plants were transferred on the low P (LP) and high P (HP) media. Images were taken 7 days after the transfer (a) and root hair length measured (b). Scale bar 0.5mm. *** Indicates q-value < 0.0001, judged by student's t-test.

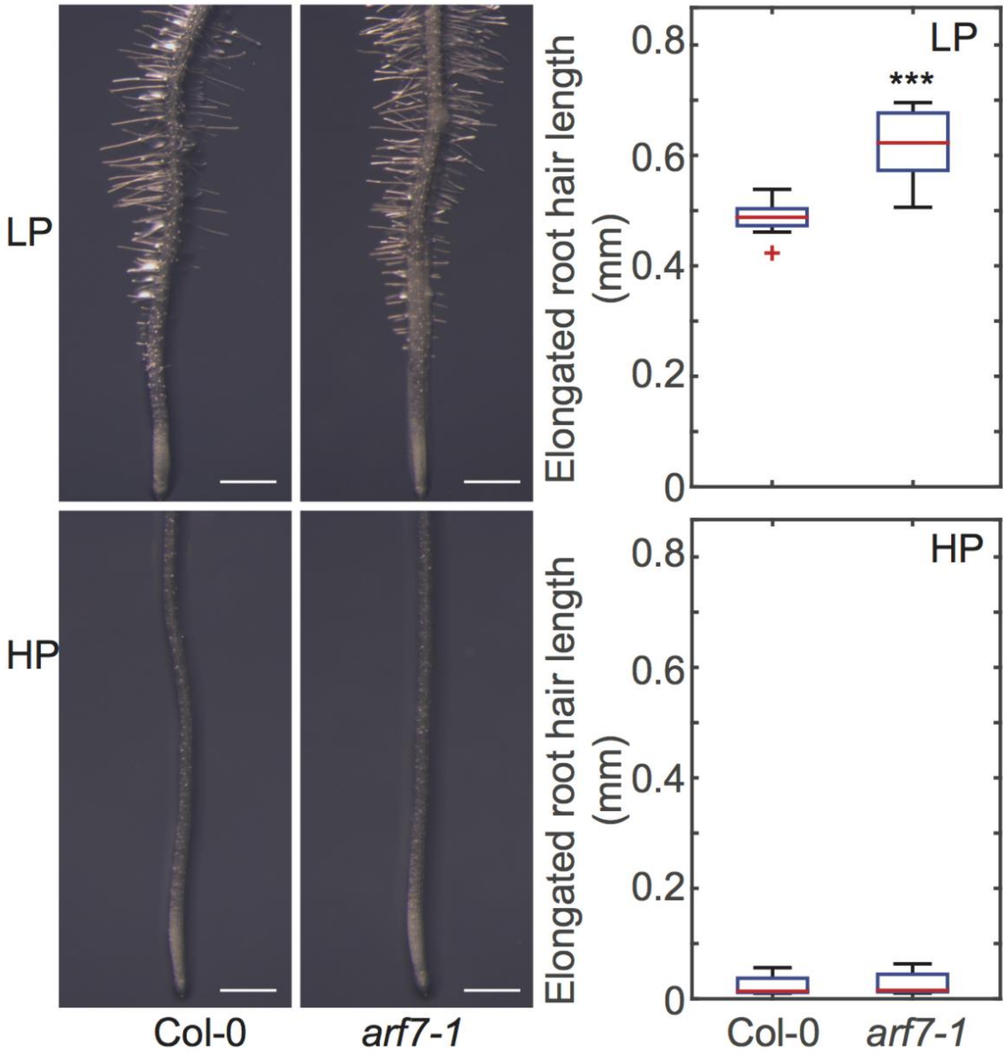

Supplementary Figure 4. *AUXIN RESPONSE FACTOR ARF7* does not regulate root hair elongation under low P in Arabidopsis.

Representative images and boxplot showing root hair growth of Columbia (Col) and *arf7-1* mutant seedlings under low P (LP) or high P (HP) conditions. *, ** and *** indicate significant difference (q-value < 0.005, 0.0001 and 0.00001, respectively) judged by Student's *t*-test. N=3, scale bar 0.5mm.

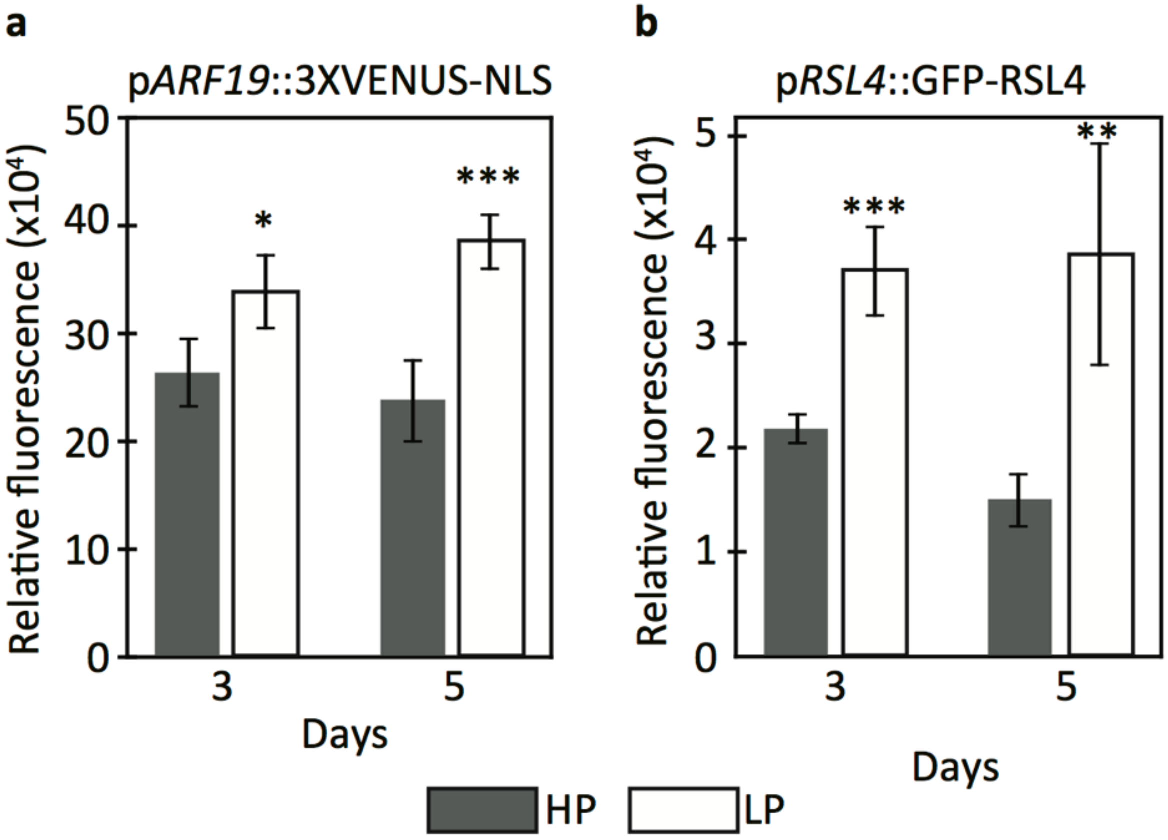

Supplementary Figure 5. AUXIN RESPONSE FACTOR *ARF19* and bHLH transcription factor *RSL4* are involved in low P responsive RH elongation in *Arabidopsis*.

Measured raw integrated fluorescence intensities of p*ARF19*::3XVENUS-NLS (a) and p*RSL4*::GFP-RSL4 (b) grown for 3 and 5 days under low (LP, empty bars) and high (HP, gray bars) P conditions. *, ** and *** indicate significant difference (q-value < 0.005, 0.0001 and 0.00001, respectively) judged by Student's *t*-test.

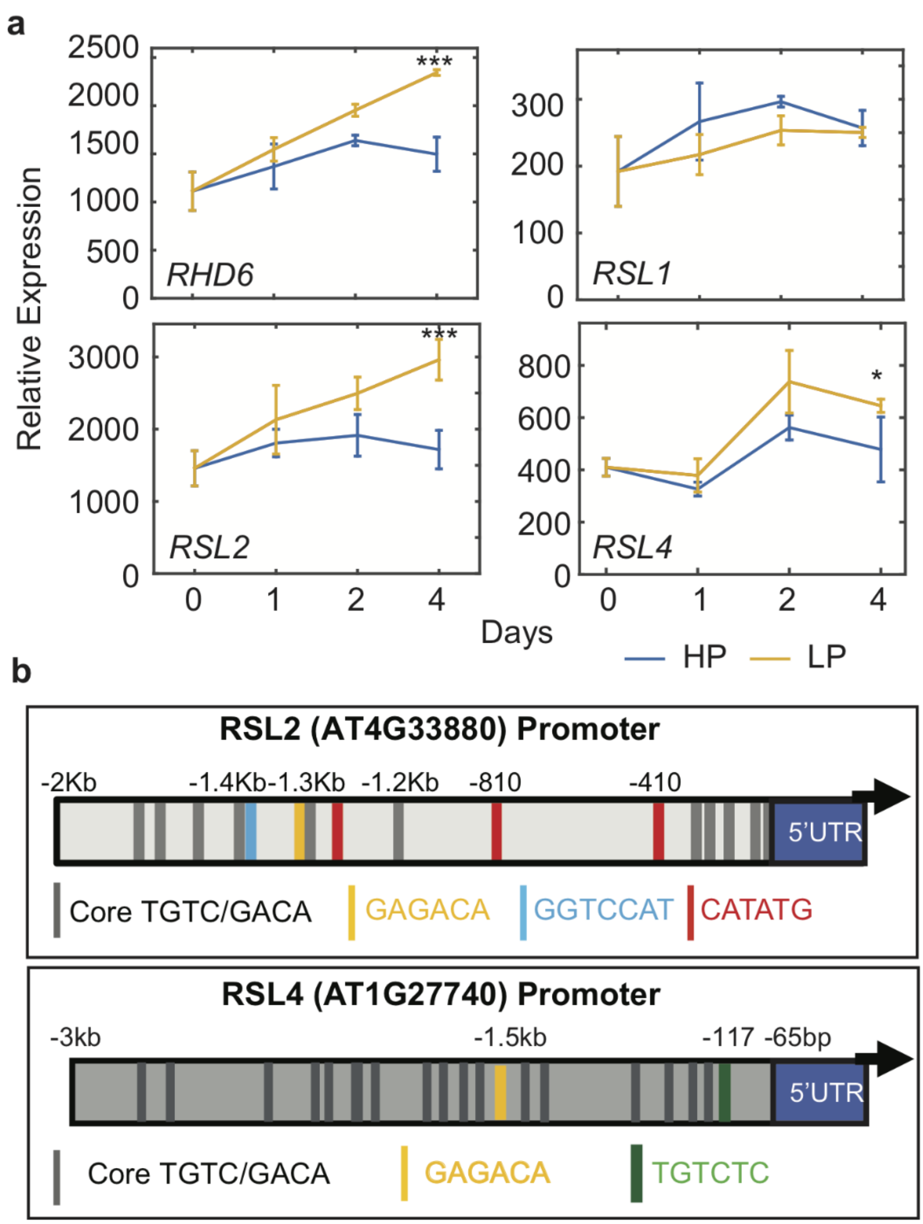

Supplementary Figure 6. *RHD6, RSL2 and RSL4* are up-regulated under low P.

a. Expression profiling studies showing bHLH transcription factors *RHD6, RSL1*, *RSL2 and RSL4* expression under low P (LP) or high P (HP) conditions. *,** and *** indicate significant difference (q-value < 0.05, 0.001, 0.0001, respectively) judged by Student's *t*-test and after Benjamini-Hochberg false discovery rate correction.

b. A cartoon showing several auxin response elements in *RSL2* and *RSL4* promoters and upstream sequence.

**Genotyping primers**	
arf19a_LP	ATCCCAATCGGCTTAAATCTC
arf19a_RP	CACCATGAGTGCTTGTGTCAC
LB3	TAGCATCTGAATTTCATAACCAATCTCGATACAC
arf19b_LP	CCGCAAATAGTCTCTTTGTGC
arf19b_RP	TCAGCACAAAGTCACAAATCG
pSKTAIL-L1	TTCTCATCTAAGCCCCCATTTGG
**RT-PCR primers**	
ARF19_RT_For	ATGAAAGCTCCATCAAATGGATTTCTTCC
ARF19_RT_Rev	CACCATGAGTGCTTGTGTCAC
**Cloning primers**	
ARF19_For	GGGGACAACTTTGTATAGAAAAGTTGGAAAATGATCCCAAAGCCTAGAGT
ARF19_Rev	GGGGACTGCTTTTTTGTACAAACTTGTTTGCTCGCTGTGTCCTTGAG

Supplementary Table 1. Primers used for genotyping and expression analysis of *arf19* mutant alleles and for the cloning of *ARF19-VENUS construct.*
